# Transition of radical, preventive and presumptive treatment regimens for malaria in China: a systematic review

**DOI:** 10.1186/s12936-020-03535-8

**Published:** 2021-01-06

**Authors:** Jian-Wei Xu, Rogan Lee, Xiao-Hong Li, Hui Liu

**Affiliations:** 1 Yunnan Institute of Parasitic Diseases, Yunnan Provincial Centre of Malaria Research, Yunnan Provincial Key Laboratory of Vector-borne Diseases Control and Research, Yunnan Institute of Parasitic Diseases Innovative Team of Key Techniques for Vector Borne Disease Control and Prevention (Developing), Training Base of International Scientific Exchange and Education in Tropical Diseases for South and Southeast Asia, Puer, 665000 China; 2 The Centre for Infectious Diseases and Microbiology, New South Wales Health Pathology, and Westmead Clinical School, The University of Sydney, Westmead Hospital, Sydney, NSW 214 Australia; 3 National Institute of Parasitic Diseases, Chinese Center for Disease Control and Prevention, Shanghai, 200025 China

**Keywords:** Malaria, Radical treatment, Prevention, Presumptive treatment, Mass drug administration, Treatment regimen, China

## Abstract

**Background:**

Globally, malaria is still a major public health challenge. Drug-based treatment is the primary intervention in malaria control and elimination. However, optimal use of mass or targeted treatments remains unclear. A variety of radical, preventive and presumptive treatment regimens have been administrated in China and a systematic review was conducted to evaluate effectiveness, and discuss experiences, limitations, and lessons learnt in relation to the use of these regimens.

**Methods:**

The search for information includes both paper documents, such as books, malaria control annals and guidelines for malaria prevention and treatment, as well as three computer-based databases in Chinese (CNKI, WanFangdata and Xueshu.baidu) and two databases in English (PubMed and Google Scholar), to identify original articles and reports associated with drug administration for malaria in China.

**Results:**

Starting from hyperendemicity to elimination of malaria in China, a large number of radical, preventive and presumptive treatment regimens had been tried. Those effective regimens were scaled up for malaria control and elimination programmes in China. Between 1949 and 1959, presumptive treatment with available anti-malarial drugs was given to people with enlarged spleens and those who had symptoms suggestive of malaria within the last 6 months. Between 1960 and 1999, mass drug administration (MDA) was given for preventive and radical treatment. Between 2000 and 2009, the approach was more targeted, and drugs were administed only to prevent malaria infection in those at high risk of exposure and those who needed radical treatment for suspected malaria. Presumptive therapy was only given to febrile patients. From 2010, the malaria programme changed into elimination phase, radical treatment changed to target individuals with confirmed either *Plasmodium vivax* or *Plasmodium ovale* within the last year. Preventive treatment was given to those who will travel to other endemic countries. Presumptive treatment was normally not given during this elimination phase. All cases of suspected were confirmed by either microscopy or rapid diagnosis tests for malaria antigens before drugs were administered. The engagement of the broader community ensured high coverage of these drug-based interventions, and the directly-observed therapy improved patient safety during drug administration.

**Conclusion:**

A large number of radical, preventive and presumptive treatment regimens for malaria had been tried in China with reported success, but the impact of drug-based interventions has been difficult to quantify because they are just a part of an integrated malaria control strategy. The historical experiences of China suggest that intervention trials should be done by the local health facilities with community involvement, and a local decision is made according to their own trial results.

## Background

Malaria still remains high on the list of diseases that causes major health burdens, globally. According to World Malaria Report 2019 from the World Health Organization (WHO), progress has slowed or even stalled. The rate of reduction of malaria mortality was slower in the period 2016–2018 than in the period 2010–2015. In 2018, an estimated 228 million cases of malaria occurred worldwide. *Plasmodium vivax* is the predominant parasite in the WHO Region of the South-East Asia, the Americas, the Eastern Mediterranean and the Western Pacific. The 53% of *P. vivax* burden is in the WHO South-East Asia Region, with the majority being in India (47%); and *P. vivax* is responsible for 75% of malaria cases in the Americas [1]. A high proportion of individuals in endemic malaria regions can be asymptomatic and have submicroscopic blood levels of parasitaemia, especially *P. vivax* [2]. The WHO plans to eliminate *Plasmodium falciparum* malaria by 2025 and turn all Greater Mekong Subregion countries into non-indigenous malaria by 2030 [3]. However, *P. vivax* malaria elimination is much more challenging than *P. falciparum* [4]. Clearance of infectious reservoir with drug-based interventions is the primary strategy for malaria control and elimination [3], but, optimal use of mass or targeted treatments remains unclear [4]. In the past, China suffered seriously from malaria hyperendemicity and epidemics [5]. Malaria transmission has been interrupted to achieve zero indigenous cases reported in China since 2017. This has been achieved by the implementation of integrated interventions for malaria control, as well as socio-economic and environmental development, such as urbanization, change of natural environment which influenced the malaria vector abundance and transmission dynamics [6].

The regimens used in China are defined as follows: a treatment regimen is a formulation, route of administration, dose, dosing interval and duration of treatment with a medicine; radical treatment usually applies to infections with either *P. vivax* or *Plasmodium ovale* and consists of medicines that treat for both blood and liver stages of the parasite to achieve complete cure; preventive treatment is the intermittent administration of a full therapeutic course of an anti-malarial either alone or in combination to prevent malarial illness by maintaining therapeutic drug levels in the blood throughout the period of greatest risk; presumptive treatment is the administration of an anti-malarial drug or drugs to people with suspected malaria without testing or before the results of blood examinations are available; mass drug administration (MDA) is the administration of anti-malarial treatment to all age groups of a defined population or every person living in a defined geographical area (except those for whom the medicine is contraindicated) at approximately the same time and often at repeated intervals [7].

A variety of radical, preventive and presumptive treatment regimens were administered through each stage of malaria control from hyperendemicity to elimination in China [8]. Mass drug administration (MDA) of radical, preventive and presumptive treatment was widely carried out to clear parasite reservoir during the early stages of malaria control (1956–1979) when malaria prevalence was high in endemic areas [8]. Since these drug regimens are just a part of the broader integrated intervention strategy of malaria control and elimination in China, their significance in achieving this goal cannot be appropriately measured. However, these regimens have helped clear parasites from endemic communities. Malaria control and elimination strategies require that clearance of infectious reservoirs is the top priority along with vector control and protection of vulnerable people. The approach of drug administration, regardless of whether malaria parasites are detected with or without the presence of clinical symptoms, might effectively contribute the reduction of malaria morbidity and mortality to reach malaria elimination status in China. In context that most of available literature on this topic from China is in Chinese, it is necessary to review literature on this topic in Chinese and then present the results of historally used radical, preventive and presumptive treatment regimens in English.

The objective of the systematic review is to report a full picture of radical, preventive and presumptive treatment regimens that have been used in China. The review will then discuss their experiences, limitation and lessons learnt, but it is not intended to document their efficacy or give any recommendations.

## Methods

### Searching strategies

The preferred reporting items for systematic review were used to select books, articles and annual malaria control reports to be included in this review. Published books, malaria control annals, manuals and guidelines for malaria prevention and treatment were manually searched from the library and archives of Yunnan Institute of parasites, and also the authors individual collections, including books, annual reports, manuals and guidelines for malaria control. A computerized systematic strategy was adopted to search articles in Chinese from CNKI, WanFangdata and Xueshu.baidu, and articles in English from PubMed and Google Scholar. Both clinical trials and control interventions were retrieved to be included in the review using the terms in both Chinese and English: China AND malaria AND radical treatment, China AND malaria AND prevention, China AND malaria AND presumptive treatment, China AND malaria AND mass drug administration. Only clinical trials and control interventions that were conducted in China were included. All books, research articles published in journals, unpublished documents such as annual reports, manuals and guidelines for malaria control before 20 December 2019 in Chinese and English language were included in this review [9].

### Inclusion criteria

The primary objective of this review was to present radical, preventive and presumptive treatment regimens for malaria that have been used in China, and their transitions in the history from malaria hyperendemicity to elimination in China. Contents on radical, preventive and presumptive treatment in books, original articles and annual malaria control reports written in Chinese and English, and conducted in China were included in this systematic review [9, 10].

### Exclusion criteria

Contents on clinical treatment for laboratory confirmed malaria patients in books, original articles and annual malaria control reports written in Chinese and English were excluded from this systematic review [9, 10].

### Data extraction and synthesis

All key contents from the selected articles were recorded in a data extraction file created in Microsoft word. The key contents extracted from each selected paper included treatment regimen, targeted population, efficacy and safety outcome and impact as well as author or agency, publication date, study design and document type, study or intervention location, sample size. The extracted data of treatment regimens were listed in a table. The main interventions, target populations and their impact were presented in each one of five phases from malaria hyperendemicity to elimination [9, 10].

### Ethical considerations

Since this is a systematic review of literature, ethical approval was not required.

## Results

### Treatment targets and regimens

In China, control and elimination of malaria were divided into five phases: (1) baseline survey and preliminary control (1949–1959),(2) epidemic control (1960–1979), (3) further reduction of malaria burden (1980–1999), (4) consolidation of achievements gained (2000–2009), and (5) elimination (2010–now) [8]. In the early stages of malaria control programmes in China, a principle of local community–based programmes was emphasized in the planning. Local control strategies were guided by the community-based health sector, who took into account the malaria burden and environmental and socio-economic conditions. The investment for malaria control in China comes from seven levels (national, provincial, prefecture, county, township, administrative village and natural village). The investment from community level (administrative village and natural village) was through the supply of labour to which no funding was given. No statistical information for the mean annual budget dedicated to combat malaria prior to 2003 was available. Both limited resources and drug availability were, therefore, key critical factors to be considered for malaria treatment prior to 2003. After this period, the Global Fund to Fight AIDS, Tuberculosis and Malaria (GFATM) provided five malaria project grants with the mean annual budget of US$8.96 million. This allowed China to purchase enough equipment, bed nets, drugs, and other supplies for malaria control in addition to funding training and supporting intervention activities.

Decision-making and transition through the various treatment phases was basically left to local governments. The higher administration authorities (i.e. national, provincial and prefecture level) delegated the responsibility to reduce malaria burdens to local government (i.e. below the prefecture or municipal level) on a yearly basis. The local governments (usually county or district level) were encouraged to reach targeted reductions, e.g. a reduction of 10% below that of the previous year by using innovative methods of control, which also included clinical trials on new treatment regimens using available registered drugs [11]. Either the county health facility or sometimes even a township hospital could decide on treatment regimens and targets prior to 2003. Regimens for radical, preventive and presumptive treatments are listed in Table 1. In order to simplify description, all dosages mentioned in this paper are for adults. The dosages for children were usually based on age in China in Table 2 [11].

**Table 1:** Regimens of radical, preventive and presumptive treatment in China (dosages shown for adults only)

Phases	Preventive treatment	Radical treatment	Presumptive treatment
Phase 1: Baseline survey and preliminary control, 1949–1959	Due to shortage of drug resources, any available antimalarial drugs such as quinacrine hydrochloride, quinine sulfate, paludrine or plasmoquine were administrated once a week during this phase [7, 12].	–	Any available antimalarial drugs were given to people with enlarged spleens and a history of malaria symptoms 6 months prior to house visit by the local health care workers [5].
Phase 2: Epidemic control, 1960–1979,	50 mg pyrimethamine once every 10–15 days [12, 14]. 50 mg cyclochloroguanidum once every 10–15 days [14]. Drug salt of 4–6.5 mg pyrimethamine daily [7, 23]. 600 mg chloroquine once every 20–30 days [7]. 300 mg chloroquine once every 7–10 day [7]. 45 mg primaquine once every 20–30 days [18]. 4 tablets of sulfadoxine–pyrimethamine (S250mg and P17.5 mg/tablet) once every 10 days [18]. 4 tablets of sulfadoxine–piperaquine (S50mg and PPQ 150 mg/tablet) once every 20 days [18]. 25 mg pyrimethamine plus 300 mg chloroquine once every 15 days [21].	50 mg pyrimethamine plus 30 mg primaquine once a month [15]. 100 mg pyrimethamine for 2 days (50 mg/d) plus 180 mg primaquine for 8 days (22.5 mg/d) [7, 16, 18]. 50 mg pyrimethamine for 2 days (50 mg/d) plus 90 mg primaquine for 4 days (22.5 mg/d) [18]. 1200 mg chloroquine for 3 days (600 mg/d1, 300 mg/d2-3 each) plus 180 mg primaquine for 8 days (22.5 mg/d) [7]. 1500 mg chloroquine for 3 days (600 mg/d1, 450 mg/d2-3 each) plus 180 mg primaquine for 8 days (22.5 mg/d) [13].	100 mg pyrimethamine for 2 days (50 mg/d) plus 90 mg primaquine for 8 days (22.5 mg/d) for mass presumptive treatment in pre-transmission season [18].
Phase 3: Further reduction of malaria burden, 1980-1999	2 tablets of sulfadoxine–pyrimethamine (SP) once every 15 days [24, 26]. 4 tablets of SP once a month [28–30, 48]. 2 tablets of SP once every 10–15 days [31]. 4 tablets of sulfadoxine–piperaquine (S-PPQ) once every 20 days [28–30]. 300 mg or 600 mg chloroquine once a week [31]. 600 mg piperaquine once a month [31]. 50 mg pyrimethamine once every 15 days [31]. 25 mg or 50 mg nivaquine once every 15 days [31].	4 tablets of SP plus 22.5 mg primaquine once a day for 4 days [27]. 1500 mg chloroquine for 3 days (600 mg/d1, 450 mg/d2-3 each) plus 90 mg primaquine for 4 days (22.5 mg/d) [31]. 1500 mg chloroquine for 3 days (600 mg/d1, 450 mg/d2-3 each) plus 180 mg primaquine for 8 days (22.5 mg/d) [31]. 180 mg primaquine for 8 days (22.5 mg/d) to clear *P. vivax* with long incubation periods [27]. 50 mg pyrimethamine once a day for 2 days plus 22.5 mg primaquine once a day for 5 days [20]. 15 mg primaquine once a day for 10 days [19].	1500 mg chloroquine for 3 days (600 mg/d1, 450 mg/d2-3 each) plus 22.5 mg primaquine once a day for 4 days in pre-transmission season [27]. 600 mg chloroquine plus 22.5 mg primaquine once in spring season [27].
Phase 4: Consolidation, 2000–2009	Targets: only people at high risk 600 mg PPQ once a month [26–31, 46]. 300 mg chloroquine once every 7-10 days [28].	Targets: only suspected malaria cases. 22.5 mg primaquine once a day for 8 days [26–31]. 1200 mg chloroquine for 3 days (600 mg/d1, 300 mg/d2-3 each) plus 180 mg primaquine for 8 days (22.5 mg/d) [26–33]. 1500 mg chloroquine for 3 days (600 mg/d1, 450 mg/d2-3 each) plus 180 mg primaquine for 8 days (22.5 mg/d) [29, 32, 33].	Targets: febrile patients 1200 mg chloroquine for 3 days (600 mg/d1, 300 mg/d2-3 each) plus 90 mg primaquine for 4 days (22.5 mg/d) [31, 32]. 600 mg chloroquine for one day in *P. vivax* endemic areas, 600 mg PPQ in *P. falciparum* and mixed endemic areas. In case of presumptive treatment worked, a standard treatment followed [27].2880 mg dihydroartemisinin (40 mg/tablet)-piperaquine phosphate (320 mg/tablet) for 3 days [13, 26, 28].
Phase 5: Elimination, 2010-	Targets: only people being travel to endemic areas of other countries 600 mg PPQ once every 30 days [41, 44, 45].	Targets: people with a history of *P.vivax* and *P. ovale* infections last year 180 mg primaquine for 8 days (22.5 mg/d) [41–45].	None recommended

**Table 2:** The age-based dosages for children in China [11]

Age rang (years)	Dosage
< 1	1/10–1/8 of adult dosage
1–3	1/6–1/4 of adult dosage
4–6	1/3 of adult dosage
7–12	1/2 of adult dosage
13–15	3/4 of adult dosage
≥ 16	Adult dosage

### Phase 1: Baseline survey and preliminary control, 1949–1959

In December 1951, the Government Administration Council published “The Work Protocol of Malaria Control in Ethnic Minority Areas” as a working protocol for malaria burden surveys and interventions. The central government mobilised health professionals to enter hyperendemic areas of ethnic minority communities in southwest China [8]. Initially, these health professionals from the central government did a baseline epidemiological survey together with recruited and trained local health staff. At the same time, control interventions were also conducted e.g. in Yunnan province, two treatment regimens were adopted: (1) presumptive treatment was given to people with enlarged spleens and clinical disease suggestive of malaria in the last 6 months; (2) the remainder of the community received weekly preventive treatment. However, data on drugs used and treatment regimens was not well documented. Early accessible data indicates that drug usage depended on what could be obtained due to poor supply during these years. The various drugs available included quinacrine hydrochloride, quinine sulfate, acrichinum, paludrine and/or plasmoquine (Table 1) [12, 13]. By 1955, China had completed the baseline survey and reduced malaria burden by about 50% in the main endemic areas across the whole country. The outcomes and information gained from early control strategies and the baseline survey helped the Ministry of Health develop “The First National Malaria Control Programme” in 1956 [8, 12].

### Phase 2: Epidemic control, 1960–1979

By 1960, China established a functional health system for malaria control. However, natural disasters and political factors challenged the effectiveness of the malaria control programme. Firstly, alternating droughts and floods created plenty of breeding sites for anopheline mosquitoes of *Anopheles sinensis* and *Anopheles anthropophagus* resulting in an epidemic of *P. vivax* in northern China in the early 1960s [8]. Secondly, The Great Cultural Revolution campaign suspended malaria interventions which led to the occurrence of epidemics in some parts including northern and southwestern China with the annual malaria incidence (API) increasing from 5.96 per 1000 in 1966 to 29.61 per 1000 person-years in the whole country in 1970 [8, 13]. Subsequently, clinical trials were undertaken to evaluate radical treatment regimens against relapse of *P. vivax* (Table 1). All of these clinical trials reported significant reduction in malaria burden [8, 11–19]. Based on the analysis of these results, the national malaria advisory committee (NMAC) recommended the treatment regimens of 1200 mg or 1500 mg chloroquine (CQ) for 3 days together with 180 mg primaquine (PQ) for 8 days for both standard clinical and radical treatments [11]. In addition, the NMAC recommended a regimen of 50 mg pyrimethamine once every 10–15 days for prevention [8, 11]. During this phase, local treatment regimens including preventive and radical treatments were used in all endemic areas of central and southern provinces of China. Normally, preventive treatment was administered during the transmission season, usually from June to September in most areas; and radical treatment was given during winter or spring, which is before or at the start of a new malaria transmission season. In hyperendemic areas, a high coverage was emphasized for both preventive and radical treatments [8, 12–23]. For example, 20–50% of a total population of 60 million people in Jiangsu Province received radical treatments and 3.3–10% of them received preventive treatments from 1973 to 1979. A total of 27,974,966 people received radical treatments in 1975 alone. These interventions reduced *P. vivax* incidence from 113.6 per 1000 person-years in 1972 to 9.0 per 1000 person-years in 1979 [23]. In five central Chinese provinces, which included Henan, Jiangsu, Shandong, Hubei and Anhui, approximately 60 million (range of 47–80 million) radical treatment regimens and 90 million (range of 66.7–124 million) preventive treatment regimens were given between 1974 and 1979. These treatment regimens reduced malaria burden by 86.06%, from 13.73 million cases in 1973 to 1.91 million cases in 1979 [8]. Yunnan Province was always an intensive transmission area due to its unique ecology and the presence of suitable vector complexes. The six species of anopheline vectors identified in this province were *An. sinensis*, *An. anthropophagus, Anopheles minimus, Anopheles dirus, Anopheles jeyporiensis* and *Anopheles kunmingensis* were identified as malaria vectors. Another two species, *Anopheles maculatus* and *Anopheles pseudowillmori* were suspected vectors. Comprehensive control measures were needed and MDA was the main approach in this province [12, 13]. Yunnan Province initially conducted a pilot trial in Menghai County on the China-Myanmar border. The trial utilized MDA with available drugs, including atebrin, pamaquine, and cyclochloroguanidum (these drugs are no longer in production) to treat a population of 17 662 for both radical and preventive treatments. The combination of indoor residual spraying with insecticides and MDA successfully resulted in no indigenous malaria infection in 1962. With the success of this trial, the whole province was included in a scaled up version of this study, thus effectively decreasing malaria burden by 86% [13].

### Phase 3: Further reducing the malaria burden, 1980–1999

At the start of this phase, MDA was still the overall approach, but focus gradually shifted from a broad coverage of the population to specific at risk communities. The number of treatment regimens dispensed gradually declined along with the reduction in the malaria burden. From 1980 to 1999, a total of 269.8 million preventive and 129.96 million radical treatment regimens were administered nationwide. These included 31.98 million radical treatment regimens to people who had a history of malaria episode in the previous year, 91.40 million preventive treatment regimens to residents in hyperendemic communities and 6.58 million preventive treatment regimens to specific populations such as migrants. As a result, this large-scale radical and preventive treatments reduced malaria burden by 99.31% and lowered the annual malaria incidence from 3378.3 per million person-years in 1980 to 23.4 per million person-years in 1999 [8]. In Yunnan Province alone, the number of malaria cases dropped from about 40 000 in 1980 to 10 000 in 1999 [13].

In 1987, a randomized controlled trial for malaria prevention was conducted on travellers crossing the China-Myanmar border [24]. Initially, a radical treatment regimen of 1500 mg CQ for 3 days together with 180 mg PQ for eight days was used to clear malaria parasites from all participants (n = 161), before the transmission season (February). Then each person in the preventive group received two tablets (50 mg, dosage for an adult) of sulfadoxine -pyrimethamine (SP) once every 15 days (Table 1). Those in the control group (n = 290) were given two vitamin C tablets each as placebo. The study reported that only one *P. falciparum* case occurred in the preventive treatment group, compared to 23 *P. falciparum* and 9 *P. vivax* cases in the control group [24].

Another two radical treatment trials were conducted to prevent relapse of the temperate strain of *P. vivax* found in high altitude areas in Yunnan Province in 1980s. The strain of *P. vivax* was recognised as having long incubation periods. In one trial, participants (n = 4871) who had a malaria episode within the last 12 months were given an adult dosage of 180 mg primaquine for 8 days (Table 1) before the transmission season. Nothing was given to the control group (n = 620). This trial reported that no cases of relapse occurred among the radical treatment group but eight (1.3%) cases were detected in the control group within 1 year [25]. In another trial, 180 mg of primaquine was administered over 8 days to the radical treatment group resulting in a relapse rate of only 1.0% (2/208) compared to 8.0% (16/201) in the control group [25]. Based on these two trials, the authors concluded that administration of 180 mg primaquine for 8 days before transmission season can prevent relapse of *P.vivax* strains with long incubation periods [25].

### Phase 4: Consolidation, 2000–2009

During this phase, the national guidelines recommended two preventive treatment regimens, 600 mg piperaquine once a month and 300 mg CQ once every 7–10 days for malaria endemic areas [26]. For radical treatment of *P.* vivax, a single regimen of 180 mg PQ for 8 days was recommended for every endemic area before the transmission season [26]. The national guidelines did not recommend any presumptive treatment regimens [26]. However, in practice, only a single regimen of 600 mg piperaquine once a month was used for prevention [27–33]. The regimen of 1200 mg CQ for 3 days plus 180 mg PQ for 8 days was used for radical treatment in central and northern China (Table 1) [34–36]. Whereas in Yunnan Province, which was considered to have high transmission intensity in remote areas, the radical treatment regimen was 1500 mg CQ for 3 days plus 180 mg PQ for 8 days, and presumptive treatment with dihydroartemisinin-piperaquine and CQ/PQ was still being administered to suspected *P. falciparum* and *P. vivax* malaria cases, respectively (Table 1) [27–33].

In 2007 a cluster intervention trial was conducted to evaluate curative efficacy of the regimen using 1200 mg CQ for 3 days together with 180 mg PQ for 8 days in Anhui Province. This trial divided 15 villages into three groups (five villages/group). In the first group, radical treatment regimen was given to individulas who had a history of malaria in the last 12 months. In the second group, the regimen was administered to the malaria cases together with other family members and those living in the neighboring households. In the third group, the treatment regimen was given to all villagers. When the annual malaria incidence data were compared between 2007 and 2006, there was a significant (P < 0. 01) decline in malaria incidence in the third group but not in the first and second groups (P > 0. 05) [37]. Following the results of this trial, the radical treatment regimen of 1200 mg CQ for 3 days plus 180 mg PQ for 8 days was scaled up to control malaria resurgence in all individuals living in Anhui and Henan Provinces. As incidence declined in the whole of China, radical treatment was scaled back to those with malaria in the last 12 months, their family members and neighbours [35–37].

Yunnan Province reported 25,070 malaria cases from 2006 to 2009, and a total of 334,316 presumptive treatment regimens (56,272 courses of dihydroartemisinin- piperaquine and 278,044 CQ/PQ) were administered to febrile patients (Table 1) [38]. Another 250,456 radical treatment regimens of 180 mg primaquine for eight days were administered against relapse of *P. vivax* malaria, and 158,601 preventive regimens with piperaquine were administered to people at high risk, e.g. those who cross the border to endemic areas of neighbouring countries [38, 39]. These multiple treatment strategies resulted in a reduction in malaria burden by 77.9% in Yunnan Province, i.e. annual parasite incidence (API) declined from 2.80 per 10,000 person-years in 2006 to 0.62 per 10,000 person-years in 2009. This dramatic decline in incidence in Yunnan Province allowed China to announce that its malaria control program could progress to elimination phase from 2010 [38].

### Phase 5: Elimination phase, since 2010

During this phase, the national guidelines recommended the use of artemisinin based combination therapy (ACT) for 3 days to treat *P. falciparum* infection and 1200 mg CQ for three days plus 180 mg PQ for 8 days for treatment of *P. vivax* and *P. oval*e infections [40]. All cases of suspected malaria were now tested either by microscopy or by rapid malaria antigen tests to confirm the diagnosis before treatment. The radical treatment regimen of 180 mg PQ for eight-days was only administered to people with confirmed *P. vivax* by Polymerase Chain Reaction (PCR) in last year. In border areas of Yunnan and mountainous areas of Hainan Province, this radical treatment regimen was also administered to family members, neighbouring household residents or travellers from malaria endemic areas to clear potential parasitic foci [38–44]. The presence of malaria in countries having common borders with Yunnan Province makes elimination of malaria more difficult in this region of China [44, 45]. In Yunnan province, 118,728 preventive treatment regimens of 600 mg piperaquine once a month reduced imported malaria from 1256 in 2011 to 388 cases in 2013 [44, 45]. The preventive treatment regimen of 600 mg piperaquine (Table 2) once a month is currently being used to prevent re-introduction of malaria into China by travellers from other endemic countries [41, 44, 45].

### Adverse side effects

In the available literature in Chinese, no record of human ethics applications and approvals for malaria treatment interventions were found. Investigations on safety of treatment regimens were also uncommon. This might be that public health benefits are much more important than that of individuals in Chinese ethical requirements. Only a few incidences of adverse side effects were reported resulting from radical, preventive and presumptive treatment. In one report, 43.3% people who received piperaquine phosphate for prevention had mild side effects of dizziness, nausea and cheek numbing (Table 3) [46]. Two other reports describe these mild adverse side effects and identified this as the reason for poor compliance to MDA of radical, preventive and presumptive treatment in Yunnan Province [47, 48]. In order to increase compliance and improve safety of the administered drugs, the intended target communities were identified, notified and encouraged to support the activity. At all stages of the drug administration, verbal consent was obtained from individuals before drugs were given. In addition, directly observed therapy by community health workers and local public health officers was used during the daily administration of medications [23]. Some adverse events were reported, such as dizziness and nausea etc. but a few severe and fatal cases did occur due to PQ-induced acute haemolysis (Table 3) [49–55].

**Table 3:** Summary of reported adverse side effects seen during drug administration of radical, preventive and presumptive treatment

Adverse side effects	No. observed	No. side effects (%)
(1) Preventive treatment: 600 mg piperaquine phosphate once a month [46]		
Mild side effects: dizziness, nausea and cheek numbing [46].	238	103(43.3%)
(2) Radical treatment: 90 mg primaquine for 4 days [49]		
Side effect: haemolysis [49].	2,529	8(0.32%)
(3) Radical cure: 150 mg primaquine for 5 days [50].		
Mild side effects: dizziness, nausea and headache [50]	853	114(13.4%)
(4) Radical treatment: 150 mg primaquine for 5 days plus 100 mg pyrimethamine for 2 days [50]		
Mild side effect: dizziness, nausea and headache [50]	1,381	264(19.1%)
(5) Radical treatment: 180 mg primaquine for 8 days plus 100 mg pyrimethamine for 2 days [51, 55]		
Serious side effects: dizziness, nausea, headache, shivering, hyperspasmia and eventually death [51]	1	1
Other side effects: varying levels of dizziness, nausea and headache [55]	857	174(20.3%)
(6) Accident: mistakenly taking 2500 mg pyrimethamine as a single dose [53]		
Serious side effects: hematocyanosis and eventual death [53]	1	1
(7) Radical treatment: primaquine 22.5 mg daily × 8 days and pyrimethamine 50 mg daily × 2 days [23]		
Haematuria, weakness, fever, appetite loss, abdominal pain/discomfort, dizziness and headache, bruising, epistaxis [23]	254,910	9 (0.0035%)
(8) Radical treatment: primaquine 30 mg daily × 4 days and pyrimethamine 50 mg daily × 2 days [23]		
Jaundice, fever, loss of appetite, weakness, dizziness, haematuria, dark coloured urine, abdominal pain, cyanosis, and headache [23]	444,589	40 (0.009%)

### An example of declining malaria from hyperendemicity to elimination in the Honghe Prefecture

**Fig. 1:**
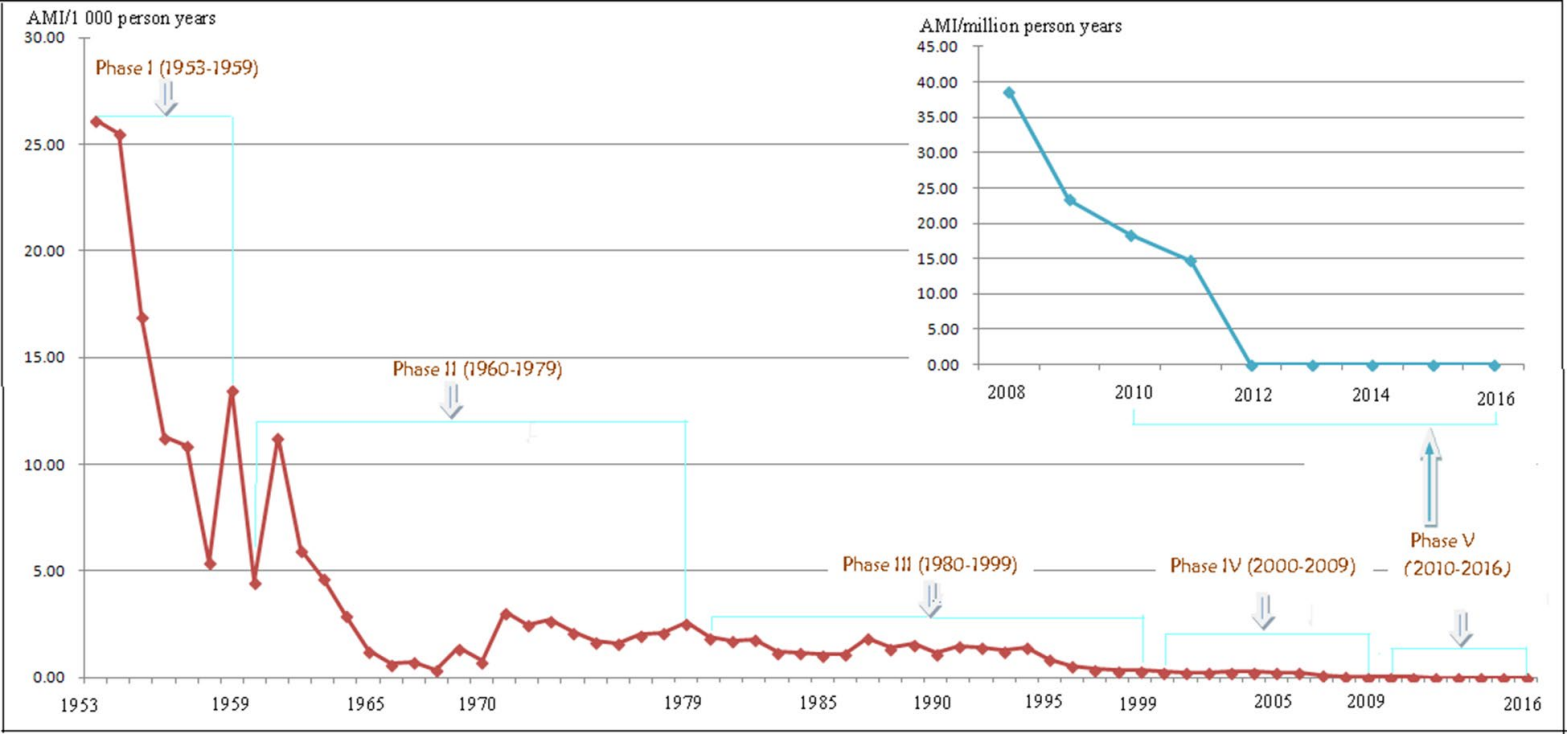
Annual malaria incidence (AMI) in Honghe Prefecture from 1952 to 2016, Yunnan Province, China. The five major phases in the malaria control and elimination program in China are indicated by the arrows

Honghe Prefecture on the China-Vietnam border with a population 4.55 million was a malaria hyperendemic area. A total 43,071 cases were recorded with an annual malaria incidence at 26.61 per 1000 person-years in 1953 [56]. The intervention of radical, preventive and presumptive treatments followed the changes in accordance with the five phases from hyperendemicity to elimination as described above. Malaria transmission was interrupted in 2012 and the Prefecture is now malaria free. According to “China National Guidelines for Malaria Elimination Evaluation and Certification”, Yunnan Provincial Health and Family Planning Commission convened a panel of experts from government health and information management departments to monitor and evaluate the laboratory’s diagnostic competency. This panel of experts reviewed epidemiological and entomological data and determined that Honghe Prefecture had achieved malaria elimination status, and certified malaria free on 4th November, 2015 [12]. This is an example of how radical, preventive and presumptive treatment, integrated intervention with vector control by indoor residual spraying (IRS) with insecticides since 1950s. After the 1980s, these treatment regimens, IRS and the addition of insecticide-treated bed nets (ITN) successfully achieved malaria elimination status from a previously hyperendemic region (Fig. 1).

### Community engagement and safety assurance

Engagement of the broader community by Government achieved high coverage of intervention of radical, preventive and presumptive treatment in China. Before the 1978 economic reforms, the People’s Commune financially and publicly supported the development of the rural cooperative medical system (RCMS), which was a branch of the primary health care system (PHCS) targeting rural areas in China. The People’s Commune notified communities through public education programmes on radical, preventive and presumptive treatment among communities before drug administration. Village health workers (VHW) and village malaria control workers (VMCW) were involved in the process of drug administration [12]. House-to-house visits every day were conducted to directly observe therapy was core to this methodology for increasing coverage, compliance and safety of MDA [12, 23]. In the daily house visits, individuals were questioned about adverse effects of the treatment taken the previous day. A decision would then be made by the VHW or VMCW on whether the next dose should be given. If there was an adverse effect reported, such as black tea coloured urine after administration of PQ in those with glucose-6-phosphate dehydrogenase (G6PD) deficiency, treatment would be stopped immediately. A proper rescue treatment followed according to the national guidelines and to the type of adverse reaction and severity of clinical symptoms [11, 26]. Those showing minor symptoms such as low grade haemoglobinuria, dosing with PQ would be discontinue, while those with more severe side effects would be given a blood transfusion. This approach ensured good coverage and safety of the treatment intervention [8, 23, 57]. Currently, with improved laboratory diagnostic capacity and only imported malaria, radical treatment in China changed to targetting individuals with confirmed *P. vivax* or *P. ovale* within the last year. Preventive treatment also changed and only travelers to other endemic countries received anti-malarial drugs. Administration of presumptive treatments is now no longer encouraged or used.

## Discussion

In 2017 malaria transmission was interrupted successfully and no local cases were detected in China [6]. This review describes use of radical, preventive and presumptive treatment regimens in China (Table 1). The strategy of integrated malaria control, including clearance of infectious reservoirs with radical and presumptive treatment, vector control of using IRS with insecticides and ITNs, and protecting vulnerable people with preventive treatment, has always been emphasized in the history from malaria endemicity to elimination [8, 11–13, 26]. Although no single study provides good evidence for the effectiveness of radical, preventive and presumptive treatment, but clearance of parasite reservoir was the primary strategy. This review demonstrates that the high coverage of these three kinds of treatment regimens used in China were necessary to achieve success.

Prevention was the first strategy of disease control in China. A large range of anti-malarial drugs have been used for prevention (Table 1). Currently, there is no local malaria transmission, but a regimen of 600 mg piperaquine given once a month is still being administered to travellers before they visit known endemic countries [58]. *Plasmodium vivax* has the largest geographic distribution amongst the human malaria species and is the dominant parasite in China [8]. Detection of *P. vivax* is not easy because parasitaemia can be much lower than *P. falciparum* and diagnostic test for liver-stage parasites of *P. vivax* is not currently available [59, 60]. Radical cure is still not easily achieved because the PQ is the only widely available drug that can kill hypnozoites of *P. vivax.* However, PQ can produce serious side-effects (haemolytic anaemia) in patients who have severe forms of G6PD deficiency [59–61]. The WHO recommends a 14-day course of PQ does not always clear *P. vivax* hypnozoites, the cure rate is usually less than 80% [59]. In China, a repetitive treatment strategy has successfully eliminated *P. vivax*. The Chinese national guideline for treatment against *P. vivax* is 1200 mg CQ for 3 days (600 mg/d1, 300 mg/d2-3 each) plus 180 mg PQ for 8 days (22.5 mg/d) for adult patients. The guidelines recommend treatment with 180 mg PQ for 8 days for radical cure prior to the next transmission season [58]. The tropical strain of *P. vivax* is relapses frequently [26] and in Yunnan Province some *P. vivax* cases may receive three or more rounds of PQ for radical cure. From 2008 to 2016, three targeted programmes on treatment were undertaken in Kachin Special Region II (KR2), Myanmar. Programme I (2008–2011) treated all confirmed, clinical and suspected cases; programme II (2012–2013) treated confirmed and clinical cases; and programme III (2014–2016) targeted confirmed cases only. Programme I (2008–2011) reduced malaria burden by 61% (95%CI 58%–74%), but a resurgence of malaria again mainly due to the increase in *P. vivax* cases during programmes II and III [62]. This finding indicates the necessity of presumptive treatment for suspected malaria cases. Based on this evidence, presumptive treatment of suspected malaria cases in China was sometimes used for travellers returning from endemic countries when parasite-based diagnosis was not available in time [26].

Artemisinin resistant *P. falciparum* has been found along the Cambodia-Thai and Thai-Myanmar borders [63], but until now resistance has not been detected along China-Myanmar border. This phenomenon may be attributed to the use of control strategies, including radical, preventive and presumptive treatment, which has reduced the number of malaria cases and the parasite’s genetic diversity [63–65]. The WHO now considers MDA as one of the strategies to control artemisinin-resistant malaria [66]. It is, therefore, worth considering the approach of MDA to clear all malaria from a targeted population to prevent development of resistance. The use either sub optimal drug dosing or use of inferior drugs may lead to anti-malarial drug resistance.

Targeted populations with no less than 70% treatment coverage were suggested to ensure success of the MDA [12]. To achieve this goal requires good planning followed up by implementation which includes detailed scheduling, adequate supplies, well trained personnel, awareness and involvement of targeted communities [67]. Strong political commitment from the central government in China ensured that delivery of the MDA was successful. Health staff delivered anti-malarial drugs to each household supported by social mobilisation to promote community engagement and compliance [8, 12, 13, 23]. Personnel for the MDA intervention included Chinese health officers and staff, village health workers, malaria control workers. In addition, local administrative officers helped to notify, mobilize and organize villagers during the programme [8, 13]. This involvement of local administrators in community mobilization increased villager’s compliance with the MDA.

A 100% effective coverage for malaria prevention and control interventions is not possible. Dr Xuezhong Li, who was the former Director of Yunnan Institute of Malaria Control and worked in malaria control for 38 years from 1954 to 1991 in China, said that for a successful program for malaria control and elimination, 30% of the success could be attributed to medication and other tools, but effective management had by far the major effect on success (70%). He said that this was called a “30% vs 70%” principle for malaria’s control and elimination. The expert panel on the malERA Refresh Consultative Panel on Health Systems and Policy Research recently released a paper indicating that even an intervention with medications at 98% efficacy would decay to just 37% effectiveness. Effectiveness declines in a malaria intervention when the country’s health and social systems are unable to implement equitable levels of quality treatment and population coverage [68]. In other words, the efficacy of radical, preventive and presumptive treatments is not just reliant on the treatment regimens alone, but also depends on effective local management of the programme with Central Government support [8, 12].

Administration of PQ in radical, preventive and presumptive treatment remains one of the greatest health risks for the patients who are G6PD deficient. The point of care site tests for G6PD deficiency was only recently available to support the treatment and elimination of malaria as advised by the WHO [69, 70]; it was however not available for China in the past 70 years. The directly observed therapy was a vital part of drug safety during this period. The literal translation of the instructions for MDA of radical and preventive treatment regimens says “Distribute drugs and water to the individual’s hand, watch them finish taking drugs and do not leave until drugs are swallowed” is an important requirement in the implementation [12, 23]. Although this approach is effective in malaria control, it may however raise some human ethical issues regarding patient’s informed consent in taking these drugs. Choice by an individual to either participate or not to participate (i.e. informed consent) is a moral obligation by organizers of the mass drug administration [71]. In healthy individuals, radical and presumptive treatment has no direct benefits and may cause drug-associated side effects. In addition, preventive treatment also has side effects. If parasite reservoirs in a population were not cleared by taking this approach, transmission of malaria would continue and malaria would remain a health risk in the community. Someone may argue that they could be screened before treatment. However, limitations in technologies, funding and workload make mass screening almost impossible [57]. Thus, the dilemma is to clear parasite reservoirs within a population with the risk of harmful side effects to individuals. The effectiveness of drug administration largely depends on coverage, so the benefits to the community could be seen to far out weigh the risks of adverse drug reactions occurring in a few individuals. In the interest of public health [71, 72], drug administration of radical, preventive and presumptive treatment was made compulsory in China with the inclusion of directly observed therapy to minimize the potential harm [73]. However, despite the use of directly observed therapy, severe adverse effects and even death resulting from PQ occur on occasions [54–59].

These experiences and lessons are reported for both historical reasons and to generate discussion on strategies that can be used to eliminate malaria in poorly resourced countries. However, there are a number of limitations existing in this review of anti-malarial drug administration. (1) Malaria is a disease of poverty. Extraneous factors such as socioeconomic development, housing improvements, drug industry development, water sanitation, deforestation, changing agricultural practices and urbanization, might have also changed malaria transmission over this long period of time [12]. This overshadows the contributions made by various phases of drug-based interventions in reducing malaria burden in China. (2) Integrated intervention strategies including parasite clearance, vector control to interrupt transmission and protection of vulnerable people have been carried in malaria control and elimination at the same time in China. Under these circumstances, it is difficult to quantify the efficacy of drug treatment regimens (e.g. preventive and presumptive treatments), but clearing potential parasite reservoirs was the top strategy for malaria control and elimination in China. Undoubtedly these drug treatment strategies did played an important role in the changes from malaria hyperendemicity to elimination, but their effect cannot be measured. What China has done might suggest that drug administration of radical, preventive and presumptive treatment could be tried as another control strategy in different settings. (3) Most referenced studies or interventions do not go into so much detail in description of methodology. Thus, in a general term some of the data was not included in some references, such as annual reports of malaria control, which were not adequately documented to show scientific value. (4) More reliable data on administration of radical, preventive and presumptive treatments shows it has worked in five central provinces with temperate climates, Henan, Jiangsu, Shandong, Hubei and Anhui. The drug administration of radical, preventive and presumptive treatment has also been carried out in tropical provinces of Yunnan and Hainan, but lacks reliable data from Hianan. However, malaria elimination in Yunnan and Hainan and the data from Yunnan might indicate that drug administration has worked in tropical climates too.

## Conclusion

In early phase of malaria control, under instructions of reducing malaria burden from the central government in China, a large number of radical, preventive and presumptive treatment regimens have been used in China. Along with socioeconomic development and environmental changes, drug administration was just a part of the integrated strategy of malaria control. With the malaria burden reduced, drug based intervention became more targeted. During later eliminating phase, only individuals who are confirmed infected with *P. vivax* or *P. ovale* within the last year receive radical treatment. Those people who are travelling to other endemic countries are given preventive treatment. The historical experiences in China suggest that intervention trials which involve local health facilities with community involvement will enhance broader cooperation and treatment coverage. This will result in reducing malaria burden in the community by removing active cases and reservoir infections allowing malaria elimination.

## Data Availability

Not applicable.

## Abbreviations

WHO: World Health Organization
MDA: Mass drug administration
GFATM: Global Fund to Fight AIDS, Tuberculosis and Malaria
API: Annual malaria incidence
NMAC: National malaria advisory committee
PQ: Primaquine
CQ: Chloroquine
ACT: Artemisinin-based combination therapy
RCMS: Rural cooperative medical system
PHCS: Primary health care system
VHW: Village health workers
VMCW: Village malaria control workers
G6PD: Glucose-6-phosphate dehydrogenase
